# Rheumatoid Arthritis: Defining Clinical and Ultrasound Deep Remission

**DOI:** 10.31138/mjr.31.4.384

**Published:** 2020-12-28

**Authors:** Corominas Hèctor, Ana Milena Millan, Cesar Diaz-Torne

**Affiliations:** Arthritis Unit, Rheumatology and Autoimmune Diseases Department, Hospital Universitari de la Santa Creu i Sant Pau, Barcelona, Spain

**Keywords:** Rheumatoid arthritis, clinical remission, ultrasound remission

## Abstract

The prognosis of patients with rheumatoid arthritis (RA) has improved substantially in the last two decades due to the appearance of biological therapies, but above all, due to the improvement in the strategy and management of the disease. Our goal in RA should be to achieve remission, or in its absence, the lowest inflammatory activity. Achieving remission will prevent from structural and functional damage highly associated with RA itself. Clinical remission is defined as the absence of significant signs and symptoms of inflammatory disease activity, as well as the abrogation of any signs of systemic inflammation. Currently, there are some controversies about remission. Which is the real remission? Which remission criteria should be used and when? Does clinical remission mean ultrasound remission? In the present review, we try to answer and put some light into it, focusing on clinical and ultrasound deep remission.

## INTRODUCTION

Rheumatoid arthritis (RA) is the most common autoimmune inflammatory arthritis. Its prognosis has improved substantially due to the appearance of biological therapies, and especially due to the improvement in the strategy and management of the disease. The concepts of therapeutic window, early treatment and “treat-to-target” have led to a radical change in the paradigm of RA. Nowadays, the objective of the treatment is clearly defined in multiple guidelines and recommendations.^1^

Our goal in RA should be to achieve remission, or in its absence, to achieve the lowest possible inflammatory activity of the disease. Achieving remission, as it is widely accepted, would prevent from structural and functional damage.^2,3^

## WHAT DO WE UNDERSTAND FOR REMISSION?

Clinical remission is defined by the absence of significant signs and symptoms of inflammatory disease activity, as well as the abrogation of any signs of systemic inflammation. There is some debate and controversy, since different definitions of remission exist, and none are considered better than the others. The proper use of the different remission indexes supports their use in the daily practice. In the present article we analyse the concept of remission, and thus, the most commonly used indexes, both clinical and by ultrasonography.

### Activity versus remission indices

The Disease Activity Score on 28 joints (DAS28) in its version with the erythrocyte sedimentation rate (ESR) is nowadays the most used activity index. The pros and cons of this index are perfectly explained in several publications.^4^ In summary, it is described that the DAS28 score derives from the following formula: DAS28 = 0.56 × NTJ + 0.28 × NSJ + 0.7 × ln (ESR) + 0.014 × overall patient’s visual analogic scale (VAS), where NTJ is the number of tender joints, NSJ the number of swollen joints, and global VAS the assessment that the patient describes of his disease activity globally (0–100). Importantly, this formula gives asymmetric values to its different components. In the total weight of the DAS28, the NTJ and the ESR are twice the value than the NSJ and the global VAS. Therefore, in clinical situations in which these two values may be altered, we should be very cautious with its interpretation.

Likewise, there is also DAS28-CRP, switching the ESR for the CRP in mg/L. This index has the same advantages and disadvantages as the DAS28-ESR, but importantly, when the values of the laboratory are clinically similar, the DAS28-CRP may result with lower scores. Comparing both DAS28, for the same result, we would need approximately 50 mg/L of CRP for 30 mm/h of ESR. Clinically, it could be accepted that a CRP of 10 mg/L is equivalent to 30 mm/h of ESR in women and 20 mm/h in men.^5^ Thus, DAS28-ESR and DAS28-CRP should not be interchangeable when confirming remission. That being said, if we use the DAS28, we will consider remission with values of 2.6 or less. In spite of this, data showing structural progression in those patients raises the question whether to use a more restrictive value (a value of less than 2.4 is considered), or the use of imaging techniques to assess remission.^6^ Nevertheless, a single DAS28 remission value, even for a period of 6 months, does not seem to be sufficient to stop the ultrasound progression. Patients should be evaluated to determine the level of residual joint inflammation, which is a key factor of progression, even if the DAS28 is lower than 2.6. In case of using the original DAS, the cut-off point to define remission would be 1.6.^7^

Historically, the first definition of remission used was the one proposed by the American College of Rheumatology (ACR) in 1981. In order to consider that a patient was in remission, they must meet at least 5 of the following criteria for at least two consecutive months: 1) Absence or less than 15 minutes of morning stiffness; 2) absence of fatigue; 3) absence of history of joint pain; 4) absence of joint pain at the exploration or pain in movement; 5) absence of joint or tendinous swelling; and 6) ESR <30 mm/h in women or 20 mm/h in men.^8^ Those criteria were surpassed for two reasons; not only were items no longer used, but mainly, because they were hardly restrictive. Despite this factor, a sort of modified criteria in which fatigue is excluded exists, and remission is defined as the achievement of 4 of the 5 remaining items.

Other remission criteria are those that use cut-off points of the indices of activity of the disease used in RA. The most used are the Simple Disease Activity Index (SDAI) and the Clinical Disease Activity Index (CDAI). These indices are the total of the direct sum of: SDAI = (NTJ28) + (NSJ28) + VAS physician (0–10) + VAS patient (0–10) + CRP (mg/dl), and the CDAI, similarly, without the CRP.^9^ A SDAI value of ≤ 3.3 and CDAI ≤ 2.8 are considered remission. Alternatively, there are also remission criteria in activity indexes based solely on the patient's reported assessment, such as PAS, PAS-II or RAPID 3 (10,11). Specifically, the RAPID 3 index (remission 0–3) includes three self-referenced measures: physical function, pain, and overall assessment. In addition to including the assessment of the patient, this index has the advantage that it can be performed and scored by the patient before the consult in a few seconds, in contrast to the 2 minutes needed to perform the DAS28 or the CDAI, avoiding unnecessary time consumption. Therefore, this index has been validated in different scenarios, comparing with composite indices, which incorporate physical exploration or analytical values. In addition, it is significantly correlated with other composite indices, such as DAS28 and CDAI.^12^ The existence of several indexes of activity and definitions of remission, the fact that they behave differently in similar populations, as well as the lack of reliability of a few of them, forced new remission criteria to be created. Because of that, in 2011, the new ACR/EULAR criteria for clinical remission of rheumatoid arthritis (Boolean approach),^13^ often used for clinical trials, were presented. The new criteria definition should 1) include tender and swollen joints and acute phase reactants, 2) predict lack of structural damage and a stable functioning, and 3) pass the OMERACT filter of truth, discrimination and feasibility. These remission criteria are of course more stringent than previous compost indices ones. ACR / EULAR definition of Remission criteria have two parts, one intended for clinical trials and the other for clinical practice. To consider remission for clinical trials the patient had to fulfil the following criteria: 1) Boolean criteria: NPJ ≤ 1, NSJ ≤ 1, CRP ≤ 1mg/dl and global VAS ≤ 1, or the SDAI remission definition (≤ 3.3). Remission for clinical practice is present including these two definitions: 1) Boolean criteria: NPJ ≤ 1, NSJ ≤ 1 and global VAS ≤ 1, and 2) index-based, with CDAI ≤2.6. These criteria have been validated both by imaging tests and by maintenance of functional capacity.^14^ According to the proportion of patients in remission in the same population studied, we can divide the remission criteria into strict and more lax. The ACR 1981 criteria and ACR/EULAR 2011 are considered strict, as well as those defined by the cut-off point of SDAI/CDAI and PAS/RAPID3, while those defined by DAS28 and the modified ACR 1981 criteria are considered more lax.

### Ultrasound remission criteria

Lillegraven et al. demonstrated, in an observational cohort, that there is radiographic progression in patients with RA in remission, either through ACR/EULAR criteria (Boolean approach), or with other simplified remission criteria (SDAI, CDAI and DAS28-CRP). Even more, there is a delay between the achievement of remission, and its effect on radiographic progression. The shorter the remission period, the more likely it is to observe mild progression rates. Thus, sustained remission is therefore the ultimate goal to prevent joint destruction and irreversible disability in RA patients. Because of that, it is essential to carry out a strict and intensive control of the disease, which entails lower rates of progression.^15,16^

Currently, it is accepted that clinical remission does not strictly mean remission by ultrasound (US), but goes beyond. The range of radiology diagnostic tools that can assess remission is limited to joint/tendon ultrasound and musculoskeletal magnetic resonance. Based on this premise, we know that subclinical synovitis exists in patients who, according to clinical criteria, are in remission. Recent work evaluating synovial samples, showed that up to 79% of patients who achieve a Boolean remission have subclinical synovitis evaluated by grey-scale US and between 32–50% present power Doppler (PD) signal, these factors being predictors of relapse and radiographic progression even in patients treated with biological therapy.^17,18^

The definition of remission by US is not yet precise, due to the amount of aspects that may have influence on it (Doppler signal, number of joints included, and which should be included).^19^ Nonetheless, US seems to play a major role as a predictor of flare. Power Doppler signal is associated with an increased risk of radiological progression. Even more, when this residual synovitis detected by ultrasound is evaluated, it is confirmed that it is very prevalent (44%) in RA in already established clinical remission. Residual synovitis detected by ultrasound is associated with a shorter duration of clinical remission and an increased risk of losing remission (HR 1.2–1.5), this being greater when ultrasound detection was performed in an early phase of remission (3–6 first months). However, for several authors, the defect is due to a lack of sensitivity of the clinical examination to detect levels of inflammation reliable by US.19

When attempting to correlate clinical indices with US findings, there are isolated studies that have shown that the remission quantified by SDAI is closer to a real absence of inflammatory US activity (absence of PD signal), than with DAS28.^20^ On the other hand, digging in the need to achieve clinical and US remission, data from 2018 confirm that patients in ultrasonographic and Boolean remission do not present radiographic progression at 12 months,^21^ while patients with activity PD (absence of remission by US) which are in clinical remission may present histopathological findings with cell proliferation and pro-angiogenic factors, suggestive of activity at the tissue level.^22^

The role of US in clinical remission in RA is described in a recent paper by Möller et al.,^23^ which summarises that: 1) ultrasound can have added value to physical examination in patients with RA in remission; 2) Doppler subclinical synovitis can predict recurrence or new flares in the short-medium term as well as progression of structural damage; and 3) ultrasonic evaluation of subclinical synovitis in patients in clinical remission should be considered (according to usual indices: DAS28, SDAI, etc.) for its predictive role on the appearance of relapses and the progression of joint damage. In addition, there are currently different US proposals that debate the number of joints that must be evaluated to confirm ultrasound remission.

The evaluation of 7 or 12 joints seems sufficient, provided that the US examination of remission includes essentially the hands.^24^ Last, but not least, introducing US progressively to take clinical decisions for the management of biological therapies,^25^ for monitoring,^26^ and even for dose optimization,^27^ seems increasingly recommended. Conversely, some systematic US treat-to-target strategy projects in early arthritis have not demonstrated better outcomes than conventional DAS28-driven strategies.^28,29^

### “The three leg” concept of remission

All that being said, the concept of remission is complex. Without a doubt, the best approach to what we have pursued as clinicians with the goal of remission, is that in the last decade, the activity of RA at the baseline and the time of initiating MTX and anti TNF + MTX has lowered high to moderate. A 2-fold increase in remission rates achieved at 6 months is because clinicians have implemented faster, more intensive and targeted strategies (T2T) to achieve remission, which undoubtedly entails a better long-term prognosis for the disease.^30^ Schett G et al. considers that the remission is a state in which more than one factor is involved. The most demanding definition would be based on three scenarios *(“the three leg”)* that should be superimposed: 1) evident absence of clinical signs of active inflammation, 2) serological definition of remission with normalization of inflammatory parameters, but above all, documented serological conversion of ACPA and RF, and 3) the remission by ultrasound image and/or magnetic resonance.^31,32^

## CONCLUSIONS

To summarise, a better validation of the US criteria for remission according to the clinical criteria should be the target which the different methods should merge to strengthen the concept of remission *“sensu stricto”*. The goal of achieving clinical remission, together with the US diagnostic methods (grey scale and power Doppler) and laboratory parameters, is now realistic, not without difficulty of agreement. If we finally achieve the definition of US remission, we would have the three aspects of remission fully covered: clinically, serologically, and ultrasonographically.
